# Parasitoid wasp venom vesicles (venosomes) enter *Drosophila melanogaster* lamellocytes through a flotillin/lipid raft-dependent endocytic pathway

**DOI:** 10.1080/21505594.2020.1838116

**Published:** 2020-10-31

**Authors:** Bin Wan, Marylène Poirié, Jean-Luc Gatti

**Affiliations:** Université Côte d’Azur, INRAE, CNRS, ISA, France

**Keywords:** Lamellocytes, endosomes, lysosomes, rafts, *Drosophila melanogaster*, venosomes, parasitoid wasps, confocal microscopy

## Abstract

Venosomes are extracellular vesicles found in the venom of *Leptopilina* endoparasitoids wasps, which transport and target virulence factors to impair the parasitoid egg encapsulation by the lamellocytes of their *Drosophila melanogaster* host larva. Using the co-immunolocalization of fluorescent *L. boulardi* venosomes and one of the putative-transported virulence factors, LbGAP, with known markers of cellular endocytosis, we show that venosomes endocytosis by lamellocytes is not a process dependent on clathrin or macropinocytosis and internalization seems to bypass the early endosomal compartment Rab5. After internalization, LbGAP colocalizes strongly with flotillin-1 and the GPI-anchored protein Atilla/L1 (a lamellocyte surface marker) suggesting that entry occurs *via* a flotillin/lipid raft-dependent pathway. Once internalized, venosomes reach all intracellular compartments, including late and recycling endosomes, lysosomes, and the endoplasmic reticulum network. Venosomes therefore enter their target cells by a specific mechanism and the virulence factors are widely distributed in the lamellocytes’ compartments to impair their functions.

## Introduction

*Leptopilina boulardi* is a parasitoid wasp that lays an egg inside the body of *Drosophila* larvae, the hatched parasitoid larvae then developing at the expense of the host. If successful, the interaction leads to the emergence of an adult wasp instead of a fly. Parasitoid wasps are important for controlling host populations in the wild, mainly other insects, and are used as biological control agents for agricultural pests [1,2]. The parasitic success of *L. boulardi* is ensured by injecting venom with the egg, which protects the egg from the immune response of the *D. melanogaster* host, the encapsulation. In this fly species, encapsulation involves a specific type of inducible hemocytes, the lamellocytes, as well as the melanization of the capsule that they form around the egg [3–8]. The venom of *L. boulardi* contains membraned vesicles of about 200 nm produced in the venom gland by an unclear mechanism, named venosomes (previously named also Virus-Like Particles; VLPs), which are stored in the wasp venom reservoir and injected with soluble venom factors in the host hemolymph [9–14]. The injection of venom impedes encapsulation by acting on the cytoskeleton of the lamellocytes [10], and we have recently shown that the purified venosomes reproduce this phenotype and alter the encapsulation response [14]. In this last study, we demonstrated that these vesicles serve as transport vehicles for potential virulence factors which may affect the cytoskeleton of the lamellocytes, inducing a change in their morphology correlated with their loss of function. Among the virulence factors transported that co-immunolocalized with the venosomes in the lamellocytes, a RhoGTPase activating protein (RhoGAP) named LbGAP is the main candidate to produce this rearrangement of the cytoskeleton. *In vitro*, LbGAP interacts with the RacGTPases Rac1 and Rac2 [10,15,16] whose invalidation in hemocytes prevents the encapsulation response [17,18]. The venosomes therefore transport putative virulence factors toward specific recipient cells, although not in the same organism in this particular case (interspecific transport). The mechanism allowing the recognition and the entry of venosomes in the lamellocytes has not been investigated yet.

External molecules, fluids, cargoes, vesicles, and even pathogens enter cells through various endocytosis pathways such as Clathrin-Mediated Endocytosis (CME) and Clathrin-Independent Endocytosis (CIE) processes which lead to the production of internal vesicles from the invagination of the plasma membrane [19–22]. The CIE mechanism includes macropinocytosis (the cell drinking pathway), the Clathrin-Independent Carriers/Glycosylphosphatidylinositol-Anchored Protein (CLIC/GPI-AP), enriched early endosomal compartment (GEEC pathway), caveolae- or flotillin-dependent endocytosis, arf6-dependent endocytosis, and other more specific receptor-dependent pathways [19,22–24]. Once released from the inner side of the plasma membrane, endocytic vesicles travel to an endosomal compartment of the cell and merge with it. The proteins and lipids of the endocytic vesicular membrane are then either returned to the plasma membrane, or transported to the late endosomes or lysosomes for degradation, depending on the cargo and the adaptor proteins involved [19,25]. In *D. melanogaster*, phagocytosis has been mainly studied in circulating macrophage-like cells named plasmatocytes [26,27], and the presence of various endocytic pathways has been demonstrated by studies on cultured cells, isolated wing discs, oocyte nurse cells, and cultured neurons and pericardial cells for selected cell-surface proteins, signaling receptors and bulky components [28–30]. One of these studies [29] analyzed some of these pathways in embryonic hemocytes (mainly plasmatocytes) of the dynamin-like shibire (*shi*) mutant fly and showed that these cells exhibit both dynamin-dependent (such as CME) and -independent endocytosis (such as GEEC). The two pathways converge in a population of Rab7-positive endosomes and subsequently target cargoes for degradation in lysosomes.

Here, we sought to decipher how the venosomes injected with wasp venom interact with the host lamellocytes and particularly to identify the endocytic pathway involved in their entry and cell distribution of their cargoes. For this, we co-immunolocalized the venosomes of *L. boulardi* or the virulence factor LbGAP with known markers of the endocytosis pathways of the plasma membrane and the endosomal compartments available for *D. melanogaster* at different times after parasitism or after injection of purified fluorescently labeled venosomes [14].

## Results/Discussion

*Leptopilina boulardi* venosomes are membrane vesicles of about 100–300 nm that target their *D. melanogaster* host lamellocytes, specific immune cells produced in response to parasitism [31,32]. Lamellocytes are large flat cells without phagocytic capacity but with many types of vesicles in their cytoplasm suggesting active endocytic and exocytic pathways [31,32], but of which very little is known. Due to their larger size and specific shape, they are easily recognized from the main hemocyte types, the plasmatocytes [14,31,32]. Here, we used two strains of *D. melanogaster*, the YR wild-type strain [33] and the mutant Hop^Tum-l^, which continuously produces a high number of lamellocytes [34,35]. These strains are unable to encapsulate the eggs of *L. boulardi* ISm due to the modification of their lamellocytes induced by the venosomes [14,33]. We also observed that micro-injection of venom or purified venosomes in *D. melanogaster* YR larvae was sufficient to induce the production of lamellocytes (see supplementary in [14]). The co-immunolocalization of venosomes (or of the transported putative virulence factor LbGAP) with the markers of endocytosis available for *Drosophila* (Table S1) was therefore tested in these two host strains, at different times after either parasitism or microinjection of purified labeled venosomes from the ISm line (timing in Table S2).

#### Clathrin-Mediated Endocytosis (CME)

**Figure 1.**
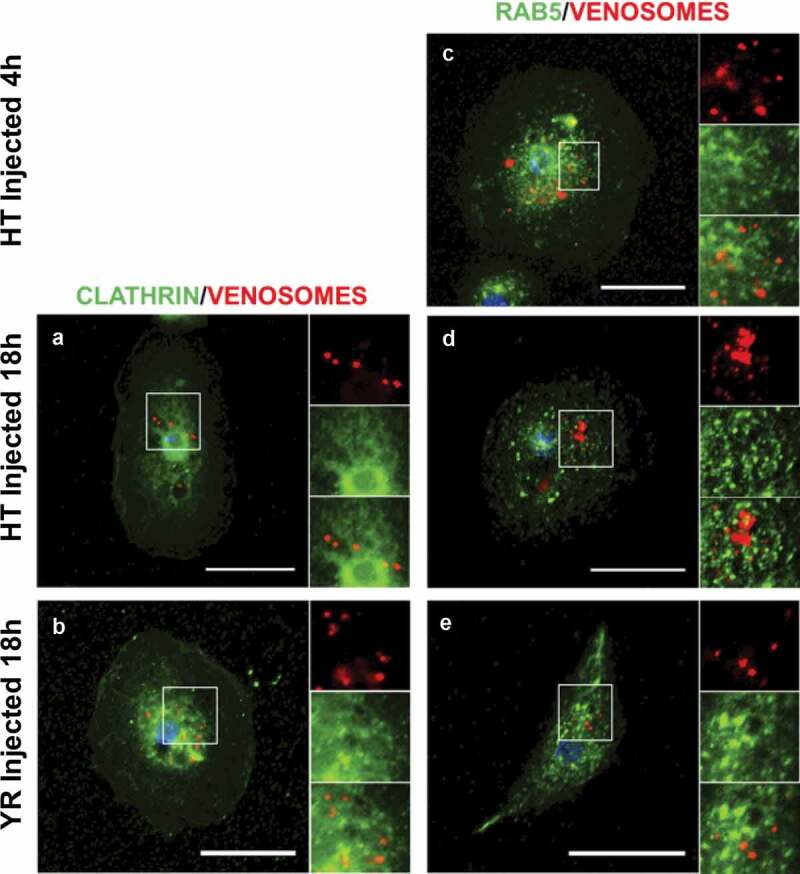
**Venosomes do not colocalize with Clathrin and Rab5 in lamellocytes**. The confocal microscopy observations of the lamellocytes 4 h or 18 h after the injection of fluorescently labeled venosomes (red) into the larvae of *D. melanogaster* YR and Hop^Tum-l^ (HT) show no colocalization with either Clathrin (a-b) or Rab5 (c-e) (Green). c, lamellocytes of Hop^Tum-l^ larvae 4 h after the injection; a and d, lamellocytes of Hop^Tum-l^ larvae 18 h after the injection; b and e, lamellocytes of YR larvae 18 h after parasitism. Bars, 20 µm

Clathrin is present in the lamellocytes of *D. melanogaster* as shown by an intense green labeling, especially around the nucleus (Figure 1). After the injection of red fluorescent venosomes in L2 larvae, no colocalization with clathrin was observed (Figure 1; see Supplementary data in Supplementary Material for the percent of co-immunolocation). Because once internalized, the vesicles uncoated from clathrin rapidly fuse with the early (sorting) endosomal compartment, we searched for co-localization with Rab5, a marker of this structure [36]. No co-localization could be found in the lamellocytes of the two *Drosophila* strains 4 h after injection nor 18 h after injection into Hop^Tum-l^ (Figure 1). Since our previous data showed that the venosomes circulate in the larval hemolymph for at least 24 h after parasitism or injection [14], they can therefore enter the lamellocytes continuously during this period. This then suggests that the lack of co-immunolocalization is not due to an experimental problem, i.e. a too rapid entry of the venosomes into the cells by CME and the rab5 co-localization missed due to the observation time. Moreover, since new lamellocytes can form during the time frame of observation [7,8], we should have seen more co-immunolocalization than that observed (see Supplementary data). Thus, the CME and the early endosomal compartment do not seem to be involved in the uptake of venosomes. Clathrin-coated vesicles are generally small (of the order of 100 nm) and cannot accommodate larger cargoes due to the curvature limitation imposed by the coat. Since the venosomes are vesicles in the range of 100–300 nm, they may not fit well in the clathrin vesicles. Other possible endocytic pathways that can uptake large vesicles/particles are macropinocytosis and endocytosis of the lipid raft domain.

#### Macropinocytosis

**Figure 2.**
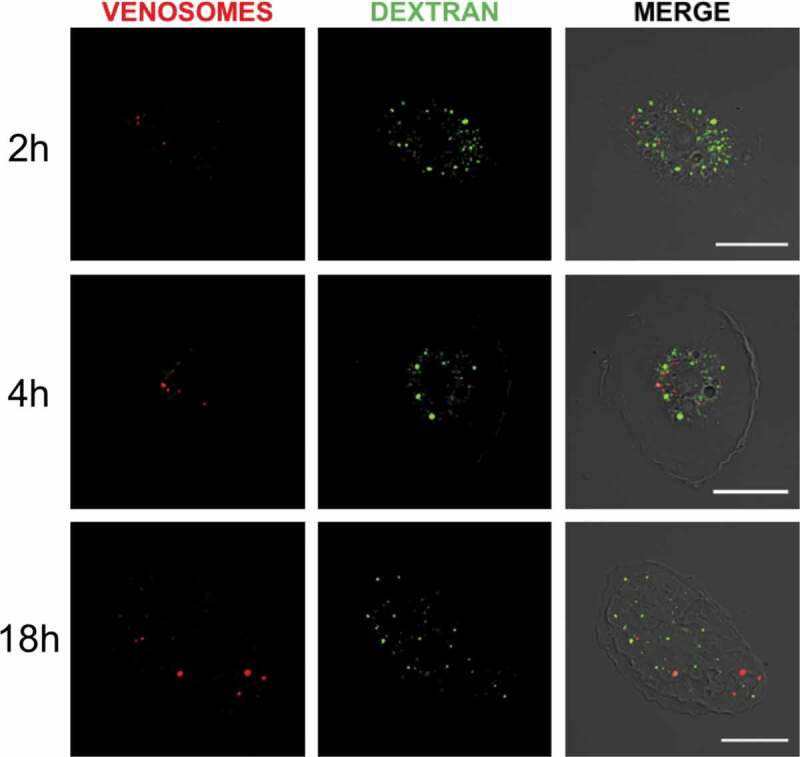
**Absence of venosomes in macropinocytic vesicles**. Internalization of the fluid phase marker Dextran (green fluorescence) by macropinocytosis and of venosomes (red) in lamellocytes of Hop^Tum-l^ larvae after co-injection (right pictures, merged of the two fluorescence channels). Few colocalization between the venosomes and dextran was observed 2 h after the injection , and very few venosomes colocalized with dextran 4 h and 18 h after the injection . Bars, 20 μm

Macropinocytosis was tested by micro-injecting a mixture of fluorescently labeled venosomes and dextran into the larvae. Dextran is a classical probe for the internalization of the fluid phase [37]. Using Hop^Tum-l^, we observed that the fluorescent dextran and the venosomes were internalized by the lamellocytes (Figure 2) but after 2 h, a very few number of colocalization was observed (about 4% of the labeled dextran spots/vesicles colocalized with venosomes, which represent about 20% of the total cell venosomes), and at 4 h and 18 h still few dextran vesicles colocalized with venosomes (7 and 8%, respectively, representing between 20% and 30% of the total cell venosomes), although in some cases dextran labeled a large part of the cell, making it difficult to ascertain a specific co-immunolocalization. The venosomes and dextran therefore seem to be sequestered in different compartments suggesting that macropinocytosis may be not the primary route of entry for the venosomes.

#### Raft-dependent endocytosis

**Figure 3.**
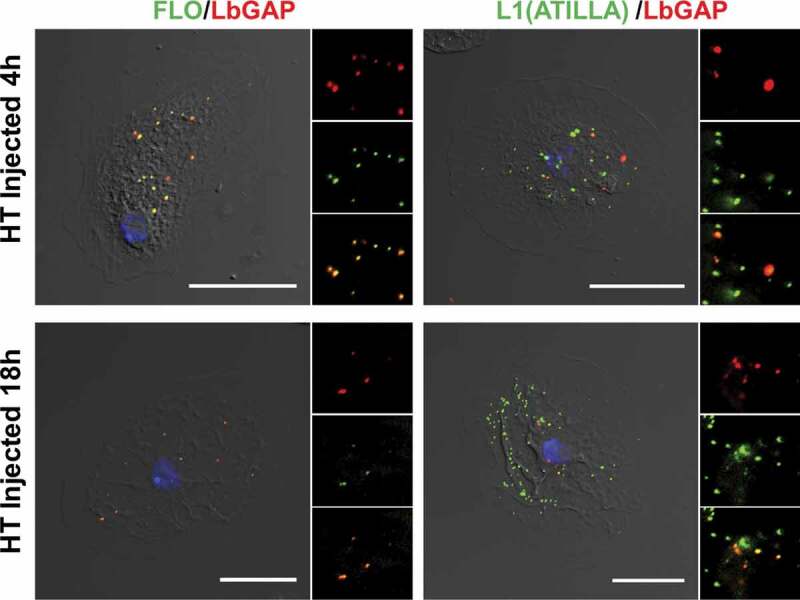
**LbGAP co-immunolocalizes with Raft membrane proteins in *Drosophila* lamellocytes**. Merged pictures of Hop^Tum-l^ (HT) lamellocytes 4 h and 18 h after injection of larvae with venosomes, fixation and immunostaining with an anti-LbGAP antibody (red) and either anti-Flotilline 1 or anti-L1/Atilla antibodies (Green) (the inserts show the individual fluorescence channel and the merged image for a selected area). The yellow/orange spots on the merged pictures indicate a co-immunolocalization between LbGAP and Flotilline 1 or L1/Atilla. Nuclei are stained with DAPI (blue). Bars, 20 µm

In mammalian cells, the main raft-dependent pathway involves the caveolae which require two types of proteins to form a vesicular coat, caveolins, and cavins [38,39]. However, there are no genes linked to caveolins and cavins annotated in the *Drosophila* genome (https://flybase.org). Another raft-dependent endocytosis is the (CLIC)/GPI-AP-enriched early endosomal compartment (GEEC pathway) which is important for internalizing and recycling GPI-anchored proteins and lipids [19,24]. This pathway gives rise to tubular endosomes rich in GPI-linked proteins and typically incorporate large volumes of extracellular fluid. Although not definitive, the fact that dextran and the venosomes did not colocalize minimizes the possible role of this pathway. A third raft-domain endocytic pathway is associated with flotillins [40]. Like mammals, *Drosophila* has two flotillins, flotillin 1 (*Flo1*) and flotillin 2 (*Flo2*) [41]. Flotillins form hetero-oligomers constitutively associated with cholesterol-enriched lipid microdomains like GPI-anchored proteins. We used here an antibody against flotillin 1 which labeled numerous small spots/vesicles in the cytoplasm of the lamellocytes (Figure 3 and Figure S1). At 4 h and 18 h after injection, almost 100% of LbGAP “spots” (used as a marker for venosomes entry) colocalized with flotillin spots (Figure 3). A similar result was obtained after parasitism of the two *Drosophila* strains (Figure S1).

The raft-dependent endocytosis also plays a role in recycling surface anchored GPI-proteins, as well as other membrane proteins such as integrins. To further investigate the specificity of this venosome-flotillin endocytosis, we took advantage of the presence of two well-described lamellocytes surface markers: one is the GPI-protein Atilla/L1 [7], the other the Myospheroid beta-integrin [42–44]. Studies in mammals have shown that flotillins mediate the endocytosis of the GPI-anchored CD59 protein and colocalize with it in early endosomes [20]. Interestingly, Atilla/L1 is part of the Ly6 protein family like the CD59 antigen of human leukocytes [44–46]. As with flotillin, the Atilla/L1 labeling showed a spot-like pattern, and almost all of the LbGAP spots colocalized with Atilla/L1 spots (Figure 3 and Figure S1) whereas myospheroid, which gave a fuzzy decoration of the cell, did not colocalize with LbGAP (Figure S1). This result is in favor of a specific flotillin-endocytic pathway for the venosomes uptake in lamellocytes, with no evidence of flotillin-dependent recycling of myospheroid.

#### Further distribution in endocytic compartments

**Figure 4.**
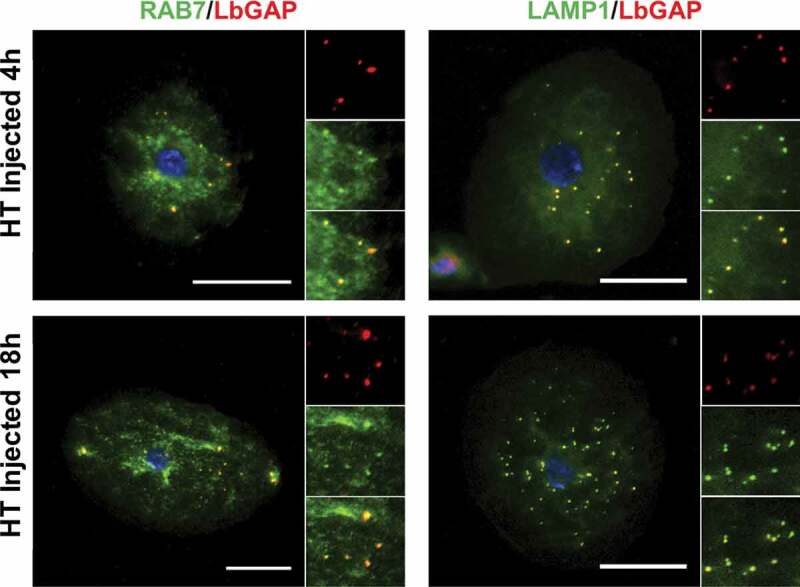
**LbGAP co-immunolocalizes with endolysosomal compartments in *Drosophila* lamellocytes**. Merged pictures of Hop^Tum-l^ (HT) lamellocytes 4 h and 18 h after injection of larvae with venosomes, fixation and immunostaining with an anti-LbGAP antibody (red) and either anti-Rab7 or anti-Lamp1 antibodies (Green) (the inserts show individual fluorescence channel and the merged image for selected areas). The yellow/orange spots on the merged pictures indicate a co-immunolocalization. Nuclei are stained with DAPI (blue). Bars, 20 µm

**Figure 5.**
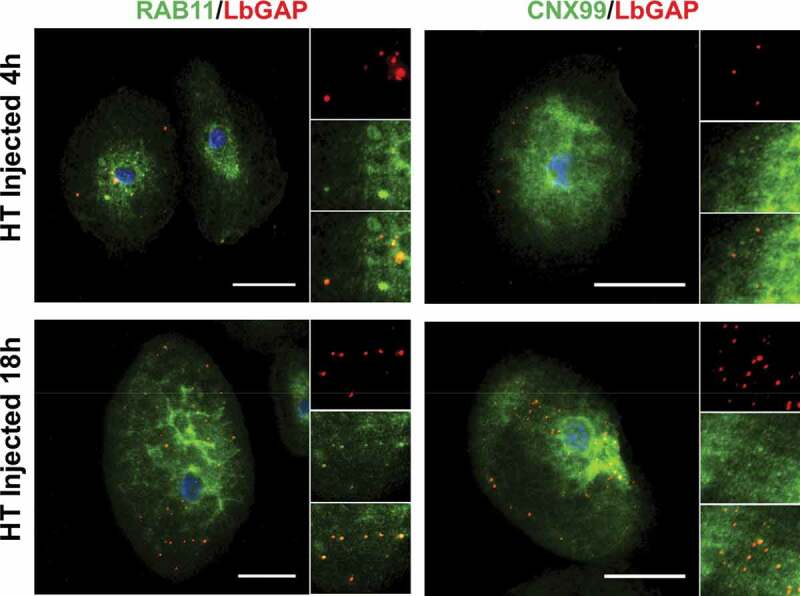
**LbGAP co-immunolocalizes with recycling endosomes and endoplasmic compartments in *Drosophila* lamellocytes**. Merged pictures of Hop^Tum-l^ (HT) lamellocytes 4 h and 18 h after injection of larvae with venosomes, fixation and immunostaining with anti-LbGAP antibody (red) and either anti-Rab11 or anti-CNX99 antibodies (Green) (inserts show each individual fluorescence channel and the merged image for selected areas). The yellow/orange spots on the merged pictures indicate a co-immunolocalization. Nuclei are stained with DAPI (blue). Bars, 20 µm

Since the venosomes, once entering the lamellocytes, were not directed to sorting Rab5 positive-endosomes, we wondered if they were only “bypassing” this step and could be retrieved in other classic endosomal compartments or if they followed a completely different and specific intracellular route. Normally, after entering the sorting endosomes, the cargoes are directed to the recycling or degradation pathways depending on the adaptor protein to which they bind and their molecular properties [21,36,47]. Endosomes sorted for degradation (endolysosomes may be the main route for raft-endocytosis) [24] are marked by their association with Rab7. Then, they merge with lysosomes, the maturation of which is signaled by the acquisition of Rab7 and lysosome-associated membrane proteins (LAMPs). The LbGAP spots clearly colocalize with Rab7 and Lamp-1 as early as 4 h after injection or parasitism (Figure 4 and Figure S2), suggesting that this venosome cargo has reached the endolysosomal compartments. In addition, we also observed the colocalization of LbGAP with calnexin-99, a marker of the endoplasmic reticulum (ER), and the recycling vesicles marker Rab11 (Figure 5 and Figure S3). The colocalization was already apparent 4 h after the injection and parasitism, suggesting that LbGAP is rapidly distributed in almost all endocytic compartments.

#### Colocalization with *Rac1*

Because LbGAP can target Rac GTPases, particularly Rac1 and Rac2 [15], to induce the rearrangement of the cytoskeleton of lamellocytes, changing their morphology from round to elongated (fusiform or bipolar) cells, we tried to co-immunolocalize this factor with Rac1. Rac1 showed intense labeling of the lamellocyte cytoskeleton (Figure S3) and although co-immunolocalization occurred in some cases, it was not a general scheme suggesting that LbGAP did not accumulate at the Rac1 location.

## Conclusion

In this study, we examined the internalization mechanism of the venosomes and their trafficking in their target lamellocyte cells. We demonstrate that the uptake of the venosomes in lamellocytes is mainly a flotillin/raft-dependent process. Venosomes quickly enter these cells and their associated LbGAP protein (a suspected virulence factor) is rapidly transported to recycling endosomes, endolysosomes, and the endoplasmic reticulum network. An exception is the lack of colocalization with the sorting compartments of Rab5 positive-endosomes. Interestingly, studies on embryonic hemocytes (mainly plasmatocytes) suggest a similar by-pass of this compartment by a dynamin-independent pathway involving raft-domains [29]. Interestingly, while larval plasmatocytes are mainly phagocytic cells [27], they also participate in the initiation of capsule formation [4]. Entry of the venosomes by an endocytic lipid raft pathway may also allow transported virulence factors to disrupt the function of these cells, a possible effect that has not been studied in detail yet. We found no discrepancies between the injection of labeled venosomes and parasitism, a more natural situation, indicating that no other component(s) of the venom is required for the cell to uptake the venosomes as has been suggested for PDVs, virus-derived ovarian vesicles used by other wasp species to produce virulence factors in host cells [48,49]. It will be interesting to test in the future whether the venom vesicles of other closely related species such as *L. heterotoma*, which can also enter the lamellocytes and induce shape changes and cell lysis, use the same endocytic pathway, and if the factors transported, which may differ from those of *L. boulardi* [50–52], show the same intracellular distribution.

We have also observed that LbGAP remains as large spots within the cells even after being released from the venosomes membrane, confirming our previous work [14,15]. LbGAP, and other transported proteins, which include another RhoGAP named LbGAP2 carrying a mutation in the active site [14,50], may form a matrix allowing a slow release of this factor or the increase of its local concentration for its action on the cell, maybe explaining why no colocalization with Rac1 could be observed at the cytoskeleton level. Another possible explanation is that this venom material scrambles the cell endocytic pathway, which requires the action of small GTPases [36,53], thereby blocking its normal function and the cycling of endosomal vesicles. This could cause cell poisoning and affect the transport of new components from the cell surface required for the capsule to form around the egg, such as myospheroid [43]. More work is needed to understand the mechanism of action of the transported factors but since flotillin/raft dependent endocytosis is not a constitutive pathway but a signal-triggered one, our work also suggests that venosomes bind to receptor(s) in the flotillin/raft domain of the plasma membrane of the lamellocytes, paving the way for its/their research.

## Materials and methods

### Biological material

The virulent ISm strain of *L. boulardi* originates from Nasrallah (Tunisia) [54] (Gif stock number G431). It was reared at 25°C on the Nasrallah *D. melanogaster* strain (Gif stock 1333) susceptible to this parasitoid. After emergence, the adults were kept at 20°C on agar medium with honey. All experiments were performed with 5–10 days-old-mated females.

The *D. melanogaster* YR strain (Gif-1088) was produced from isofemale lines from a population of Brazzaville (Congo). This strain is homozygous for the resistant allele of a major gene for resistance to the ISy line of *L. boulardi*, located in a region of chromosome 2 R [55]. The Hop^Tum-l^ mutant strain was obtained from the Bloomington *Drosophila* stock center (stock number 8492). *Drosophila* strains were reared on standard medium (10% cornmeal, 10% yeast, agar, and nipagine) at 25°C.

### Venosomes purification and microinjection with Dextran

The venosomes were obtained by dissection of the reservoirs of *L. boulardi* ISm females. Twenty to 30 reservoirs were dissected in 20–30 µl of Insect Ringer (IR; KCl, 182 mM; NaCl, 46 mM; CaCl_2_, 3 mM; Tris-HCl, 10 mM). The venom extract was centrifuged 5 min. at 500 g at 4°C to remove large debris and the supernatant 10 min at 15,000 g at 4°C. The pellet containing the venosomes [13,14] was resuspended in IR and centrifuged again. This last pellet was diluted in 50 µl IR and labeled with NHS-Alexa Fluor 680 (2 mM; Interchim) as described previously [14]. After one IR washing to remove the non-reactive NHS-Alexa, the fluorescent venosomes were used directly or mixed with fluorescently labeled 10 kDa Dextran-Alexa fluor 488 (10 µg/µl; In Vitrogen). The labeled venosomes or the venosomes-dextran solution were microinjected into second-instar host larvae (FemtoJet, Eppendorf).

### Parasitism

For immunohistochemistry of hemocytes, batches of 30 second instar larvae (L2) placed on a dish with *Drosophila* food were submitted to parasitism by three female wasps for 4 h [33]. After this period, the parasitoids were removed, and the larvae left at 25°C until used. For each batch, three larvae were dissected and only those batches in which three larvae contained an egg or a parasitoid larva were used for the hemolymph collection.

### Lamellocytes immunolabelling and imaging

The hemolymph of *Drosophila* larvae was collected in a drop of 30 µl of PBS at the end of the parasitism period and 14 h later, or 2 h, 4 h, and 18 h after microinjection with labeled venosomes or labeled venosomes-Dextran solution. The drop was placed in the center of a 15-mm glass coverslip in a 24-well culture plate and the hemocytes were allowed to adhere for 1 h.

For macropinocytosis , observations were made on live cells to keep the dextran within the cell compartments. Adherent cells were washed with PBS and the coverslip was mounted on a slide using a 5 μl drop of PBS. Although PBS is not the best medium to keep cells alive, we were able to observe and image the fluorescent probes in less than 20 minutes before cell degradation starts. The number of venosomes that co-immunolocalized with a green fluorescent vesicle was counted in three different cells from three separate experiments for the three times.

For immunostaining, adherent cells were washed with PBS and fixed with 4% formaldehyde in PBS for 15 minutes at room temperature (RT). After three washes with PBS, the cells were permeabilized with PBS-0.1% Triton X100 for 20 min then incubated with PBS-0.3% BSA for 30 min and with the different primary antibodies diluted in the same buffer for 1 h at RT. After three PBS washes, they were incubated with the secondary antibodies for 1 h at RT. After three additional PBS washes and one with deionized water, the coverslip was mounted on a slide using an antifading medium containing DAPI to stain the cell nucleus (Interchim). The commercial antibodies used are described in Table S1, the anti-LbGAP was described previously [56], all were used at a 1:500 dilutions. All antibody solutions were prepared in PBS 0.3% BSA. Secondary antibodies were fluorescently labeled goat anti-rabbit IgG (Fluoprobes 547 H for LbGAP (Interchim), 1/1,000, and Andy Fluor 488 for other rabbit polyclonals (GeneCopoeia), 1/500), goat anti-mouse IgG (Andy Fluor 488, GeneCopoeia, 1/500) and rabbit anti-goat IgG (FITC, Sigma, 1/500).

The preparations were observed and imaged using a laser scanning confocal microscope equipped with an array scan detector (LSM 880; Zeiss) and the pictures were edited with the Zen software (Zeiss). Each experiment was repeated at least twice. We choose to display the picture of the cells that have a high number of venosomes to clearly show the co-immunolocalization. The percentage of co-immunolocalization between venosomes or LbGAP and the indicated marker was estimated for each experiment by counting the spots on the cells of at least three different samples (supplementary Table S3).

## Supplementary Material

Supplemental Material

## References

[1] Pennacchio F, Strand M. Evolution of developmental strategies in parasitic Hymenoptera. Annu Rev Entomol. 2006;51:233–258. 16332211 10.1146/annurev.ento.51.110104.151029

[2] Van Lenteren J, Bolckmans K, Köhl J, et al. Biological control using invertebrates and microorganisms: plenty of new opportunities. BioControl. 2017;63:39–59.

[3] Carton Y, Kitano H. Changes in the hemocyte population of *Drosophila* larvae after single and multiple parasitization by *Cothonaspis* (Parasitic Cynipidae). J Invertebr Pathol. 1979;34:88–89.

[4] Russo J, Dupas S, Frey F, et al. Insect immunity: early events in the encapsulation process of parasitoid (*Leptopilina boulardi*) eggs in resistant and susceptible strains of *Drosophila*. Parasitology. 1996;112:135–142. 8587797 10.1017/s0031182000065173

[5] Carton Y, Poirié M, Nappi AJ. Insect immune resistance to parasitoids. Insect Sci. 2008;15:67–87.

[6] Poirié M, Carton Y, Dubuffet A. Virulence strategies in parasitoid Hymenoptera as an example of adaptive diversity. C R Biol. 2009;332:311–320. 19281961 10.1016/j.crvi.2008.09.004

[7] Honti V, Csordás G, É K, et al. The cell-mediated immunity of *Drosophila melanogaster*: hemocyte lineages, immune compartments, microanatomy and regulation. Dev Comp Immunol. 2014;42:47–56. 23800719 10.1016/j.dci.2013.06.005

[8] Kim-Jo C, Gatti J-L, Poirié M. *Drosophila* cellular immunity against parasitoid wasps: a complex and time-dependent process. Front Physiol. 2019;10:e1005746–8. 10.3389/fphys.2019.00603 PMC6529592 31156469

[9] Dupas S, Brehelin M, Frey F, et al. Immune suppressive virus-like particles in a *Drosophila* parasitoid: significance of their intraspecific morphological variations. Parasitology. 1996;113:207–212. 8811846 10.1017/s0031182000081981

[10] Labrosse C, Carton Y, Dubuffet A, et al. Active suppression of *D. melanogaster* immune response by long gland products of the parasitic wasp *Leptopilina boulardi*. J Insect Physiol. 2003;49:513–522. 12770630 10.1016/s0022-1910(03)00054-4

[11] Morales J, Chiu H, Oo T, et al. Biogenesis, structure, and immune-suppressive effects of virus-like particles of a *Drosophila* parasitoid, *Leptopilina victoriae*. J Insect Physiol. 2005;51:181–195. 15749103 10.1016/j.jinsphys.2004.11.002

[12] Gueguen G, Rajwani R, Paddibhatla I, et al. VLPs of *Leptopilina boulardi* share biogenesis and overall stellate morphology with VLPs of the *heterotoma* clade. Virus Res. 2011;160:159–165. 21704090 10.1016/j.virusres.2011.06.005 PMC3905611

[13] Gatti J-L, Schmitz A, Colinet D, et al. Diversity of virus-like particles in parasitoids’ venom: viral or cellular origin? In: Beckage NE, Drezen J-M, editors. Parasitoid viruses: symbionts or pathogens. Elsevier; 2012. p. 181–192. DOI:10.1016/B978-0-12-384858-1.00015-1.

[14] Wan B, Goguet E, Ravallec M, et al. Venom atypical extracellular vesicles as interspecies vehicles of virulence factors involved in host specificity: the case of a *Drosophila* parasitoid wasp. Front Immunol. 2019;10:1688. 31379874 10.3389/fimmu.2019.01688 PMC6653201

[15] Colinet D, Schmitz A, Depoix D, et al. Convergent use of RhoGAP toxins by eukaryotic parasites and bacterial pathogens. PLoS Pathog. 2007;3:e203. 18166080 10.1371/journal.ppat.0030203 PMC2156102

[16] Colinet D, Schmitz A, Cazes D, et al. The origin of intraspecific variation of virulence in an eukaryotic immune suppressive parasite. PLoS Pathog. 2010;6:e1001206. 21124871 10.1371/journal.ppat.1001206 PMC2991256

[17] Williams MJ. Rac1 signaling in the *Drosophila* larval cellular immune response. J Cell Sci. 2006;119:2015–2024. 16621891 10.1242/jcs.02920

[18] Fauvarque M-O, Williams MJ. *Drosophila* cellular immunity: a story of migration and adhesion. J Cell Sci. 2011;124:1373–1382. 21502134 10.1242/jcs.064592

[19] Doherty GJ, McMahon HT. Mechanisms of endocytosis. Annu Rev Biochem. 2009;78:857–902. 19317650 10.1146/annurev.biochem.78.081307.110540

[20] Kumari S, MG S, Mayor S. Endocytosis unplugged: multiple ways to enter the cell. Cell Res. 2010;20:256–275. 20125123 10.1038/cr.2010.19 PMC7091825

[21] Kaksonen M, Roux A. Mechanisms of clathrin-mediated endocytosis. Nat Rev Mol Cell Biol. 2018;3:e03970. 10.1038/nrm.2017.132 29410531

[22] Sandvig K, Kavaliauskiene S, Skotland T. Clathrin-independent endocytosis: an increasing degree of complexity. Histochem Cell Biol. 2018;150:107–118. 29774430 10.1007/s00418-018-1678-5 PMC6096564

[23] Kirkham M, Parton RG. Clathrin-independent endocytosis: new insights into caveolae and non-caveolar lipid raft carriers. Biochem Biophys Acta Mol Cell Res. 2005;1745:273–286. 10.1016/j.bbamcr.2005.06.002 16046009

[24] Mayor S, Parton RG, Donaldson JG. Clathrin-independent pathways of endocytosis. Cold Spring Harb Perspect Biol. 2014;6:a016758. 24890511 10.1101/cshperspect.a016758 PMC4031960

[25] Maldonado-Báez L, Williamson C, Donaldson JG. Clathrin-independent endocytosis: A cargo-centric view. Exp Cell Res. 2013;319:2759–2769. 23954817 10.1016/j.yexcr.2013.08.008 PMC4157725

[26] Charroux B, Royet J. Elimination of plasmatocytes by targeted apoptosis reveals their role in multiple aspects of the *Drosophila* immune response. Proc Natl Acad Sci USA. 2009;106:9797–9802. 19482944 10.1073/pnas.0903971106 PMC2700997

[27] Melcarne C, Lemaitre B, Kurant E. Phagocytosis in *Drosophila*: from molecules and cellular machinery to physiology. Insect Biochem Mol Biol. 2019;109:1–12. 30953686 10.1016/j.ibmb.2019.04.002

[28] Narayanan R, Ramaswami M. Endocytosis in *Drosophila*: progress, possibilities, prognostications. Exp Cell Res. 2001;271:28–35. 11697879 10.1006/excr.2001.5370

[29] Guha A, Sriram V, Krishnan KS, et al. Shibire mutations reveal distinct dynamin-independent and -dependent endocytic pathways in primary cultures of *Drosophila* hemocytes. J Cell Sci. 2003;116:3373–3386. 12857788 10.1242/jcs.00637

[30] Fischer JA, Eun SH, Doolan BT. Endocytosis, endosome trafficking, and the regulation of *Drosophila* development. Annu Rev Cell Dev Biol. 2006;22:181–206. 16776558 10.1146/annurev.cellbio.22.010605.093205

[31] Rizki TM, Rizki RM. Lamellocyte differentiation in *Drosophila* larvae parasitized by Leptopilina. Dev Comp Immunol. 1992;16:103–110. 1499832 10.1016/0145-305x(92)90011-z

[32] Ribeiro C, Brehélin M. Insect haemocytes: what type of cell is that? J Insect Physiol. 2006;52:417–429. 16527302 10.1016/j.jinsphys.2006.01.005

[33] Dubuffet A, Colinet D, Anselme C, et al. Variation of *Leptopilina boulardi* success in *Drosophila* hosts: what is inside the black box? Adv Parasitol. 2009;70:147–188. 19773070 10.1016/S0065-308X(09)70006-5

[34] Hanratty WP, Dearolf CR. The *Drosophila* Tumorous lethal hematopoietic oncogene is a dominant mutation in the hopscotch locus. Mol Gen Genet. 1993;238:33–37. 8479437 10.1007/BF00279527

[35] Luo H, Rose P, Roberts T, et al. The Hopscotch Jak kinase requires the Raf pathway to promote blood cell activation and differentiation in *Drosophila*. Mol Genet Genomics. 2002;267:57–63. 11919715 10.1007/s00438-001-0632-7

[36] Donaldson JG, Johnson DL, Dutta D. Rab and Arf G proteins in endosomal trafficking and cell surface homeostasis. Small GTPases. 2016;7:247–251. 27416526 10.1080/21541248.2016.1212687 PMC5129904

[37] Lim JP, Gleeson PA. Macropinocytosis: an endocytic pathway for internalizing large gulps. Immunol Cell Biol. 2011;89:836–843. 21423264 10.1038/icb.2011.20

[38] Bastiani M, Parton RG. Caveolae at a glance. J Cell Sci. 2010;123:3831–3836. 21048159 10.1242/jcs.070102

[39] Parton RG, Collins BM. Unraveling the architecture of caveolae. Proc Nat Acad Sci USA. 2016;113:14170–14172. 27911845 10.1073/pnas.1617954113 PMC5167180

[40] Meister M, Tikkanen R. Endocytic trafficking of membrane-bound cargo: A flotillin point of view. Membranes (Basel). 2014;4:356–371. 25019426 10.3390/membranes4030356 PMC4194039

[41] Galbiati F, Volonté D, Goltz JS, et al. Identification, sequence and developmental expression of invertebrate flotillins from *Drosophila melanogaster*. Gene. 1998;210:229–237. 9573373 10.1016/s0378-1119(98)00064-x

[42] MacKrell AJ, Blumberg B, Haynes SR, et al. The lethal myospheroid gene of *Drosophila* encodes a membrane protein homologous to vertebrate integrin beta subunits. Proc Natl Acad Sci USA. 1988;85:2633–2637. 3128792 10.1073/pnas.85.8.2633 PMC280052

[43] Xavier MJ, Williams MJ. The Rho-family GTPase Rac1 regulates integrin localization in *Drosophila* immunosurveillance cells. PLoS One. 2011;6:e19504. 21603603 10.1371/journal.pone.0019504 PMC3095607

[44] Hijazi A, Masson W, Auge B, et al. *boudin* is required for septate junction organization in *Drosophila* and codes for a diffusible protein of the Ly6 superfamily. Development. 2009;136:2199–2209. 19502482 10.1242/dev.033845

[45] Lee PY, Wang JX, Parisini E, et al. Ly6 family proteins in neutrophil biology. J Leukoc Biol. 2013;94:585–594. 23543767 10.1189/jlb.0113014

[46] Tanaka K, Diekmann Y, Hazbun A, et al. Analysis of expression pattern diversification in the recently expanded insect Ly6 gene family. Mol Biol Evol. 2015;32:1730–1747. 25743545 10.1093/molbev/msv052 PMC4476152

[47] Rab SH. GTPases as coordinators of vesicle traffic. Nat Rev Mol Cell Biol. 2009;10:513–525. 19603039 10.1038/nrm2728

[48] Strand MR, Burke GR. Polydnavirus-wasp associations: evolution, genome organization, and function. Curr Opin Virol. 2013;3:587–594. 23816391 10.1016/j.coviro.2013.06.004

[49] Gauthier J, Drezen J-M, Herniou E. The recurrent domestication of viruses: major evolutionary transitions in parasitic wasps. Parasitology. 2017;145:713–723. 28534452 10.1017/S0031182017000725

[50] Colinet D, Deleury E, Anselme C, et al. Extensive inter- and intraspecific venom variation in closely related parasites targeting the same host: the case of *Leptopilina* parasitoids of *Drosophila*. Insect Biochem Mol Biol. 2013;43:601–611. 23557852 10.1016/j.ibmb.2013.03.010

[51] Goecks J, Mortimer NT, Mobley JA, et al. Integrative approach reveals composition of endoparasitoid wasp venoms. PLoS One. 2013;8:1–14. 10.1371/journal.pone.0064125 PMC3662768 23717546

[52] Heavner ME, Ramroop J, Gueguen G, et al. Novel organelles with elements of bacterial and eukaryotic secretion systems weaponize parasites of *Drosophila*. Curr Biol. 2017;27:2869–2877.e6. 28889977 10.1016/j.cub.2017.08.019 PMC5659752

[53] Qualmann B, Mellor H. Regulation of endocytic traffic by Rho GTPases. Biochem J. 2003;371:233–241. 12564953 10.1042/BJ20030139 PMC1223314

[54] Dupas S, Boscaro M. Geographic variation and evolution of immunosuppressive genes in a Drosophila parasitoid. Ecography. 1999;22:284–291.

[55] Poirié M, Frey F, Hita M, et al. *Drosophila* resistance genes to parasitoids: chromosomal location and linkage analysis. Proc R Soc Lond B. 2000;267(1451):1417–1421. 10.1098/rspb.2000.1158 PMC1690685 10983825

[56] Labrosse C, Stasiak K, Lesobre J, et al. A RhoGAP protein as a main immune suppressive factor in the *Leptopilina boulardi* (Hymenoptera, Figitidae)-*Drosophila melanogaster* interaction. Insect Biochem Mol Biol. 2005;35:93–103. 15681220 10.1016/j.ibmb.2004.10.004

