# Supplementary material for: Parasitoid wasp venom vesicles (venosomes) enter Drosophila melanogaster lamellocytes through a flotillin/lipid raft-dependent endocytic pathway
Source: Virulence. 2020 Oct 31;11(1):1512–21. doi: 10.1080/21505594.2020.1838116 (PMC7605353; doi:10.1080/21505594.2020.1838116)
Supplement: Supplemental Material [file KVIR_A_1838116_SM7075.docx]

**Supplementary Materials (Figures S1-S3)**

**Figure S1. LbGAP co-immunolocalizes only with Raft membrane proteins in *Drosophila* lamellocytes after parasitism.** Merged pictures of lamellocytes fixed and immunostained with anti-LbGAP antibody (red) and either anti-Flotilline-1 (A-C), anti-L1/Atilla (D-F) or anti-Myospheroid (G-I) antibodies (Green) (inserts show each individual fluorescence channel and the merged image for the selected area). The yellow spots on the merged picture (A-I) indicate a co-immunocalization of LbGAP and Flotilline-1 or L1/Atilla. LbGAP does not colocalize with Myospheroid. A, D, and G, lamellocytes of Hop^Tum-l^ larvae 4h after parasitism; B, E, and H, 18h after parasitism; C, F and I, lamellocytes of YR larvae 18h after parasitism. Nucleus were stained with DAPI (blue). Bars, 20µm.

**Figure S2. LbGAP co-immunolocalizes with Rab7 and Lamp-1 in *Drosophila* lamellocytes after parasitism.** A-F merged images of lamellocytes fixed and immunostained with anti-LbGAP antibodies (red) and either anti-Rab 7 (A-C) or anti-Lamp-1 (D-F) antibodies (Green) (inserts show each individual fluorescence channel and the merged image for the selected area). The yellow spots indicate co-immunocalization. A and D, lamellocytes of Hop^Tum-l^ (HT) larvae 4h after parasitism; B and E, 18h after parasitism; C and F, lamellocytes of YR larvae 18h after parasitism. Nucleus were stained with DAPI (blue). Bars, 20µm.

**Figure S3. LbGAP colocalizes with Rab11 or Calnexin99, but not with Rac1.** (A-I) merged images of fixed lamellocytes immunostained with anti-LbGAP antibodies (red) and either anti-Rab11 (A-C), anti-Calnexin 99 (D-F) or anti-Rac1 (G-I) antibodies (Green) (inserts show each individual fluorescence channel and the merged image for the selected area). Yellow spots indicate co-immunocalization of LbGAP and Rab11 or Calnexin99. LbGAP does not colocalize with Rac1. A, D and G, lamellocytes of Hop^Tum-l^ (HT) larvae 4h and B, E and H, 18h after parasitism; C, F and I, lamellocytes of YR larvae 18h after parasitism. Nucleus were stained with DAPI (blue). Scale bar, 20µm.

**Supplementary Materials (Tables S1-S3)**

**Table S1: Antibodies used**

| **Primary antibodies** | **Publication** | **Reseller** | **Secondary antibodies** |
| --- | --- | --- | --- |
| Rab 7 | Riedel et al., 2016 | DSHB  (Rab7-s) | Goat anti-mouse IgG (Andy Fluor 488, GeneCopoeia; 1/500) |
| Rab 11 | Steiner et al., 2002 | BD Biosciences  (Cat N^o^: 610656) |  |
| calnexin 99 | Riedel et al., 2016 | DSHB  (Cnx99A 6-2-1) |  |
| Flotilline-1 | Volonte et al., 1998 | BD Biosciences  (Cat N^o^: 610820) |  |
| L1/Atilla | Honti et al., 2009 | Gift from Dr. I. Ando |  |
| Myospheroid | Brower et al., 1984 | DSHB  (CF.6G11) |  |
| Rac-1 | Kuroda et al., 1996 | BD Biosciences  (Cat No: 612652) |  |
| Lamp-1  Rat Fluorescently labelled (Alexa-488) | Fujita et al., 2017 | Santa Cruz Biotechnology  (Cat N^o^: sc-19992) | none |
| Rab 5 | Huang et al., 2010 | Abcam  (Cat N^o^: ab31261) | Goat anti-rabbit IgG (Andy Fluor 488, GeneCopoeia; 1/500) |
| Clathrin | Zhang et al., 1998 | Sigma  (Cat N^o^: 031M4798) | Rabbit anti-goat IgG (FITC, Sigma; 1/500) |

Riedel, F., Gillingham, A.K., Rosa-Ferreira, C., Galindo, A., and Munro, S. (2016). An antibody toolkit for the study of membrane traffic in *Drosophila* melanogaster. *Biology Open,* *5*, 987–992.

Steiner P, Floyd Sarria JC, Glauser L, Magnin S, Catsicas S, et al. (2002). Modulation of receptor cycling by neuron-enriched endosomal protein of 21 kD. *J Cell Biol,* 157: 1197–1209.

Volonte D, Galbiati F, Li S, Nishiyama K, Okamoto T, et al. (1999). Flotillins/cavatellins are differentially expressed in cells and tissues and form a hetero-oligomeric complex with caveolins in vivo. *J Biol Chem*, 274: 12702–12709.

Honti, V., Kurucz, E., Csordás, G., Laurinyecz, B., Márkus, R., Andó, I. (2009). *In vivo* detection of lamellocytes in *Drosophila melanogaster*. Immunology Letters, 126(1-2), 83-84. https://dx.doi.org/10.1016/j.imlet.2009.08.004

Brower, D.L., Wilcox, M., Piovant, M., Smith, R.J. and Reger, L.A. (1984). Related cell-surface antigens expressed with positional specificity in *Drosophila* imaginal discs. *PNAS USA*, *81*(23), pp.7485-7489.

Kuroda, S., Fukata, M., Kobayashi, K., Nakafuku, M., Nomura, N., Iwamatsu, A. and Kaibuchi, K. (1996). Identification of IQGAP as a putative target for the small GTPases, Cdc42 and Rac1. *J Biol Chem*, *271*(38), pp.23363-23367.

Fujita, N., Huang, W., Lin, T.H., Groulx, J.F., Jean, S., Kuchitsu, Y., Koyama-Honda, I., Mizushima, N., Fukuda, M. and Kiger, A.A. (2017). Genetic screen in *Drosophila* muscle identifies autophagy-mediated T-tubule remodeling and a Rab2 role in autophagy. *Elife*, *6*, p:e23367.

Huang, H. R., Chen, Z. J., Kunes, S., Chang, G. D., and Maniatis, T. (2010). Endocytic pathway is required for *Drosophila* Toll innate immune signaling. *PNAS USA*, *107*(18), 8322-8327.

Zhang, B., Koh, Y.H., Beckstead, R.B., Budnik, V., Ganetzky, B. and Bellen, H.J. (1998). Synaptic vesicle size and number are regulated by a clathrin adaptor protein required for endocytosis. *Neuron*, *21*(6), pp.1465-1475.

DHSB: Developmental Studies Hybridoma Bank, University of Iowa, Department of Biology

**Table S2 : Experimental design with the *Drosophila* strains with the timing of the observations after parasitism or microinjection and the antibodies tested.**

|  | ***D. melanogaster* Hop^Tum-l^** | | | | ***D. melanogaster* YR** | | | |
| --- | --- | --- | --- | --- | --- | --- | --- | --- |
|  | *PARASITISM* | | *INJECTION* | | *PARASITISM* | | *INJECTION* | |
|  | **4h** | **18h** | **4h** | **18h** | **4h*** | **18h** | **4h*** | **18h** |
| **Clathrin** |  |  |  | **+** |  |  |  | **+** |
| **RAB5** |  |  | **+** | **+** |  |  |  | **+** |
| **FLOTILLINE** | **+** | **+** | **+** | **+** |  | **+** |  |  |
| **Atilla/L1** | **+** | **+** | **+** | **+** |  | **+** |  |  |
| **RAB7** | **+** | **+** | **+** | **+** |  | **+** |  |  |
| **LAMP1** | **+** | **+** | **+** | **+** |  | **+** |  |  |
| **RAB11** | **+** | **+** | **+** | **+** |  | **+** |  |  |
| **CNX99** | **+** | **+** | **+** | **+** |  | **+** |  |  |
| **MYS** | **+** | **+** |  |  |  | **+** |  |  |
| **RAC1** | **+** | **+** |  |  |  | **+** |  |  |

*After 4h there is few induced lamellocytes in the hemolymph in *D. melanogaster* YR.

**Supplementary data**

**Percent of co-immunolocation observed in the different experiments.**

The fluorescent spots of LbGAP (for parasitized conditions) or venosomes (injected conditions) were counted. Thereafter co-immunolocalized spots obtained on the stack image were counted. Percent = [(number of co-immunolocalizing spots / number of LbGAP or venosomes spots) X 100]. From 10 to 90 spots from 3 different cells from 3 different preparations (9 cells total) were counted and the mean of co-immunolocalization percentages are indicated for the different experiments.

For *Drosophila melanogaster* YR

| YR 18h parasitised | |
| --- | --- |
| FLO | 99% |
| ATILLA | 98% |
| RAB7 | 98% |
| LAMP1 | 100% |
| RAB11 | 100% |
| CNX99 | 97% |
| Myospheroid | 8% |
| RAC1 | 4% |

| YR 18h injected | |
| --- | --- |
| Clathrin | 4% |
| RAB5 | 3% |

For *Drosophila melanogaster* Hop^tuml^

| HT 4h parasitized | |
| --- | --- |
| FLO | 100% |
| ATILLA | 96% |
| RAB7 | 99% |
| LAMP1 | 100% |
| RAB11 | 99% |
| CNX99 | 70% |
| Myospheroid | 2% |
| RAC1 | 2% |

| HT 18h parasitized | |
| --- | --- |
| FLO | 100% |
| ATILLA | 96% |
| RAB7 | 99% |
| LAMP1 | 100% |
| RAB11 | 99% |
| CNX99 | 98% |
| Myospheroid | 6% |
| RAC1 | 4% |

| HT 4h injected | |
| --- | --- |
| RAB5 | 1% |
| FLO | 100% |
| ATILLA | 99% |
| RAB7 | 100% |
| LAMP1 | 100% |
| RAB11 | 100% |
| CNX99 | 94% |

| HT 18h injected | |
| --- | --- |
| Clathrin | 0% |
| RAB5 | 0% |
| FLO | 100% |
| ATILLA | 100% |
| RAB7 | 100% |
| LAMP1 | 100% |
| RAB11 | 100% |
| CNX99 | 99% |
